# A Diagnostic Pitfall of Transthoracic Echocardiography: A Case of a Missed Large Mitral Valve Thrombus in the Setting of Suspected Nonbacterial Thrombotic Endocarditis

**DOI:** 10.7759/cureus.35495

**Published:** 2023-02-26

**Authors:** Cameron Kahn, Azeem Rathore, Timothy Lasseter, William M Kogler, Emil Missov

**Affiliations:** 1 Medicine, University of Florida College of Medicine – Jacksonville, Jacksonville, USA; 2 Internal Medicine, University of Florida College of Medicine – Jacksonville, Jacksonville, USA; 3 Department of Cardiology, University of Florida Health Jacksonville, Jacksonville, USA

**Keywords:** intracardiac thrombus, cardioembolic stroke, transthoracic and transesophageal echocardiography, libman-sacks endocarditis, nonbacterial thrombotic endocarditis (ntbe), marantic endocarditis, mitral valve thrombus, sterile valvular vegetation

## Abstract

Transthoracic echocardiography (TTE) is frequently utilized in the initial evaluation of cardioembolic stroke. However, the diagnostic utility of TTE is often operator-dependent, and in conjunction with anatomical limitations, there is a range of sensitivities reported in the literature specifically in the evaluation of nonbacterial thrombotic endocarditis (NBTE). Thus, relying on TTE findings to rule out NBTE in the setting of cardioembolic stroke evaluation can lead to misdiagnosis in the absence of confirmatory transesophageal echocardiography (TEE).

We present a case of a 67-year-old female with a past medical history of hypertension, diabetes mellitus, human immunodeficiency virus (HIV), and recurrent ischemic strokes who was referred by her neurologist for TEE. Despite an initial TTE with a bubble study showing no evidence of intra-atrial septum, left ventricular thrombus, or any valvular pathology, there remained high suspicion of a cardioembolic source due to the bi-hemispheric presentation of the patient's previous strokes. Prior electrocardiography and cardiac event monitor showed normal sinus rhythm. Her TEE revealed a large, dense thrombus measuring 1.0 x 0.8 centimeters involving the anterior mitral valve leaflet with associated moderate mitral regurgitation. The patient was placed on systemic anticoagulation and discharged home with outpatient follow-up with cardiology. Our case highlights the diagnostic pitfalls of TTE use in the evaluation of cardioembolic stroke with a particular emphasis on NBTE in addition to discussing the rationale for follow-up TEE when TTE is otherwise unrevealing.

## Introduction

Cardioembolic sources account for 15-40% of all ischemic strokes with an associated 50% mortality after three years [1]. Transthoracic echocardiography (TTE) remains the modality of choice during the initial diagnostic screening tool for suspected valvular or septal defects, and although there are well-documented data on the sensitivity and specificity of echocardiography for the identification of infective endocarditis vegetations, left atrial or left ventricular thrombus, and thrombosis of prosthetic valves, there is sparse reporting on the efficacy of identifying nonbacterial thrombotic endocarditis (NBTE). Thus, we present a case that highlighted the diagnostic drawback of TTE in not visualizing a mitral valve thrombus that a follow-up transesophageal echocardiography (TEE) successfully identified. In conjunction with the patient's history, it was highly suspected that the missed mitral valve thrombus was consistent with NBTE which is a rare disorder characterized by sterile, thrombotic vegetations of the heart valves.

## Case presentation

**Figure 1:**
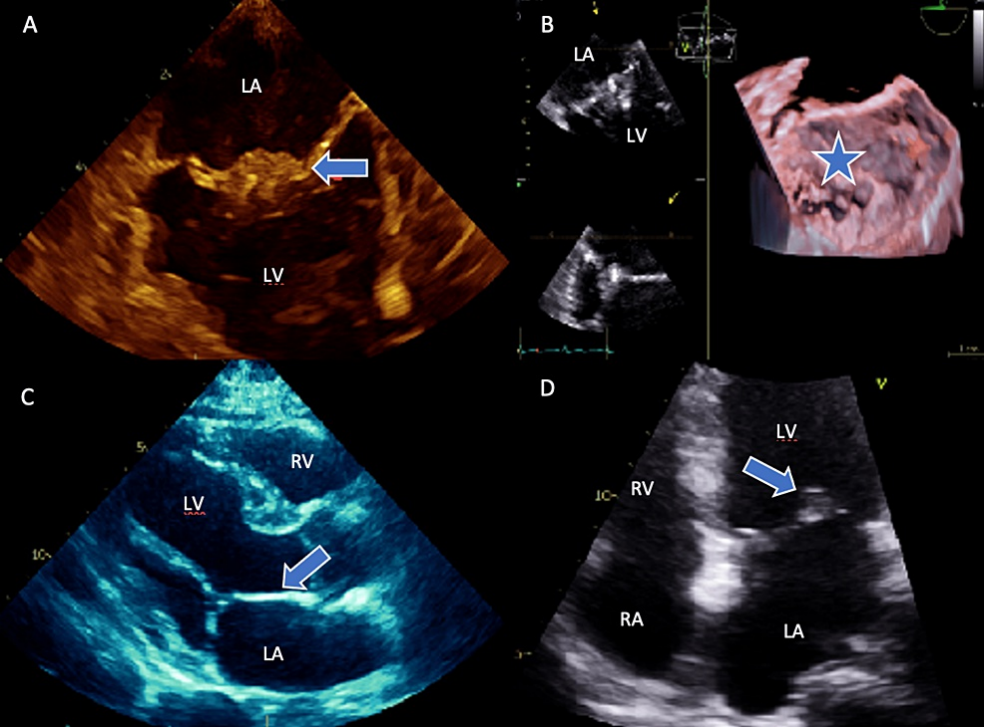
Transesophageal echocardiography with thrombus identified (panels A & B) in comparison to absent findings on transthoracic echocardiography (panels C & D) Panel A- TEE in commissural view showing a large 1.0 x 0.8 cm heavily dense mass consistent with thrombus located on the atrial side of the anterior mitral valve leaflet (arrow). Panel B- TEE 3-D image showing a large echogenic density located on the anterior leaflet of the mitral valve (star). Panel C- TTE in parasternal long axis view which shows no mitral valve pathology (arrow showing anterior mitral valve leaflet with no thrombus). Panel D- TTE in apical four chamber view which shows mildly thickened anterior leaflet of the mitral valve (arrow), otherwise no vegetation or thrombus. TTE: Transthoracic echocardiography, TEE: Transesophageal echocardiography, LA: left atrium, LV: left ventricle, RA: right atrium, RV: right ventricle

A 67-year-old female with a past medical history of hypertension, diabetes mellitus, human immunodeficiency virus (HIV), and recurrent ischemic strokes was referred by her neurologist for TEE. The only abnormality seen on the initial TTE was a thickened anterior mitral valve leaflet on apical four chamber view (Video 1); however, suspicion remained high for a cardioembolic source due to the bi-hemispheric presentation of her strokes. Of note, prior electrocardiography and cardiac event monitor showed normal sinus rhythm. Her TEE subsequently revealed a large, dense echogenic material measuring 1.0 x 0.8 centimeters located on the anterior mitral valve (Figure 1). The patient was not exposed to recent antibiotics and blood cultures were negative from prior admission; thus, the mitral valve mass was suggestive of a sterile vegetation. In addition, moderate eccentric mitral regurgitation was also present, involving the anterior mitral valve leaflet. The structural cardiology team recommended not pursuing trans-catheter thrombectomy due to an increased risk of cerebral vascular events. Additionally, cardiothoracic surgery did not recommend surgical intervention due to the patient's limited post-surgical rehabilitation potential, as she was wheelchair dependent due to hemiparesis from her prior stroke events. The patient was started on therapeutic heparin and bridged to warfarin with a goal international normalized ratio (INR) of 2-3. She was discharged home with the goal of outpatient cardiology monitoring, including both a prothrombotic workup and a repeat TEE to follow thrombus resolution, but unfortunately was lost to follow-up.

**Video 1:** TTE apical four chamber view Thickened anterior mitral valve leaflet. no vegetation or thrombus noted. TTE: Transthoracic echocardiography

## Discussion

NBTE is a rare complication in patients with prothrombotic disease states with high mortality. Due to its physical characteristics and underlying pathophysiology, terms such as “NBTE,” “marantic,” “verrucous,” and “thrombotic” endocarditis have been used interchangeably to describe the same complication [2]. The most common presentation in NBTE is stroke accounting for 59.5% of cases with an associated one-year mortality of 38%, which encourages prompt and timely identification and appropriate treatment for such lesions [3]. Indeed, due to a lack of physical examination findings specific to NBTE, clinicians must exclusively rely on echocardiography to guide management; however, as evident in our case, there remains a diagnostic blind spot, so to speak, with TTE screening.

From autopsy reports, NBTE vegetations are composed of sterile platelets and fibrin thrombi, with the prevailing pathophysiology believed to be that the thrombophilic state leads to the development of platelet/fibrin vegetation [2]. Risk factors for NBTE include systemic inflammatory conditions such as malignancy (40.5%), systemic lupus erythematosus (33.3%), and antiphospholipid antibody syndrome (35.7%) [3]. The mean age at diagnosis is 54 ± 14.5 years, with the majority of cases occurring in females (66.7%) as well [3]. Importantly, NBTE is not to be confused with culture-negative infective endocarditis, which is often due to previous antibiotic use that suppresses adequate bacterial growth on blood cultures. As for our patient, we had a 67-year-old female without a history of malignancy or autoimmune disease but was previously diagnosed with HIV. A C-reactive protein (CRP) level was not ascertained but her HIV status was near undetectable while on antiretroviral therapy. Blood cultures were negative which ruled out septic emboli. No recent antibiotic use was reported, ruling out culture negative infectious endocarditis. It is well established in literature the risk of venous thromboembolism (VTE) in uncontrolled HIV or on a protease inhibitor such as indinavir; however, our patient was nearly undetectable with a CD4 count >900 cells/mm^3^ and not on a protease inhibitor [4]. Although less likely, HIV related or undiagnosed malignancy as the cause for the sterile mitral valve vegetation could not be excluded. After being started on systemic anticoagulation, the patient was discharged with a prothrombotic workup to be conducted, including repeating her autoimmune labs, such as antinuclear antibody (ANA) titers, anti-double-stranded DNA (anti-dsDNA), lupus anticoagulant, Factor V Leiden, Protein C, and S deficiency. To confirm the diagnosis of intra-cardiac thrombus, repeat imaging would be indicated after being on anticoagulation to see if there was partial or complete resolution of the mitral valve vegetation; however, the patient was lost to follow-up at the three-month visit.

As previously alluded to, NBTE is likely underreported since the diagnosis relies exclusively on echocardiography visualizing the lesion(s). In fact, the sensitivity and specificity of TTE fluctuate based on the location of the thrombus itself. The sensitivity of TTE for detecting left ventricular thrombosis ranges between 92% and 95%, with a specificity of 86% to 88% [5]. If the thrombus is in the left atrium (LA), the sensitivity of TTE is low [6]. In addition to the anatomical limitations, it is visually challenging to differentiate between vegetation, thrombus, fibroelastoma, or the spectrum of NBTE, thus leading to the possibility of misdiagnosis [7]. Indeed, Zmaili et al. estimated that TTE is only diagnostic in 45.2% of cases compared to 97.1% of cases when TEE was utilized, and even when thrombus is detected via TTE, the extent of thrombus formation remains inferior to TEE [1,3]. Several factors affect the diagnostic yield of TTE, including vegetation size; Erbel et al. estimate that only 25% of vegetations < 5 mm and 70% of those between 6-10 mm are successfully identified [8]. The chances of finding an intracardiac thrombus are increased if there is concomitant atrial fibrillation (AF), ventricular dyskinesia, or mitral stenosis [9]. The most common valves involved were mitral at 61.9%, followed by the aortic valve at 23.8% [3]. In our case, the patient was in normal sinus rhythm without previous cardiac history and underwent initial TTE with a bubble study that was unrevealing for an intracardiac source for her stroke. However, due to high clinical suspicion, she was referred for TEE, which uncovered a large dense echogenic material consistent with a thrombus located on the anterior mitral valve that was suggestive of the rarely diagnosed NBTE.

## Conclusions

TTE is widely considered the gold-standard imaging modality for the evaluation of cardiac sources of embolic stroke, and yet we presented a case that provides a nuanced reminder of the diagnostic pitfall of over-reliance in detecting intracardiac pathology, specifically as it relates to uncovering NBTE. Ultimately, this case provides a cautionary reminder for healthcare providers to consider a follow-up TEE if the initial TTE is unrevealing during cardioembolic stroke evaluation when strong suspicion remains that a cardiac etiology remains elusive.
